# The influence of breast density and key demographics of radiographers on mammography reporting performance – a pilot study

**DOI:** 10.1002/jmrs.486

**Published:** 2021-05-24

**Authors:** Maram Alakhras, Dana S. Al‐Mousa, Alaa K. Alqadi, Haneen A. Sabaneh, Ruba M. Karasneh, Kelly M. Spuur

**Affiliations:** ^1^ Faculty of Applied Medical Sciences Jordan University of Science and Technology Irbid Jordan; ^2^ School of Dentistry and Health Sciences Charles Sturt University Wagga Wagga NSW Australia

**Keywords:** Breast, mammography, reporting, screening

## Abstract

**Introduction:**

A high demand has been placed on radiologists to perform screen reads due to higher number of women undergoing mammography. This study aims to examine radiographer performance in reporting low compared with high‐mammographic density (MD) images; and to assess the influence of key demographics of Jordanian radiographers on their performance.

**Methods:**

Thirty mammograms with varied MD were reported by 12 radiographers using the Breast Imaging‐Reporting and Data System (BI‐RADS). Radiographer performance was measured using sensitivity, specificity, positive (PPV) and negative predictive values (NPV), and area under the receiver operating characteristic curve (ROC AUC). Performance measures were compared between cases with low‐ and high‐MD and between subgroups of radiographers according to key demographics.

**Results:**

All performance measures were significantly higher in low‐ compared to high‐MD cases (*P* value < 0.0). The mean sensitivity, specificity, PPV, NPV and ROC AUC were 0.58, 0.68, 0.67, 0.63 and 0.69 respectively. PPV was significantly different for readers who had different years of experience in mammography, hours and cases per week *P* value = 0.023, 0.01, 0.017 respectively. ROC AUC was significantly different for radiographers with different number of hours and cases performed per week (*P* value = 0.001 and 0.004 respectively).

**Conclusions:**

The results of this pilot study are encouraging however a more extensive study is required to determine if Jordanian radiographers are capable of successfully taking part in breast screen reading. The lack of skills and knowledge required for correct and consistent reporting of high‐MD images highlights the need for any formal training in mammographic interpretation to focus on the dense breast.

## Graphical abstract

This study aims to assess the influence of key demographics of Jordanian radiographers and mammographic density and on radiographers’ performance in reporting mammograms. Performance of radiographers is lower in high‐mammographic breast density cases compared with low‐density breasts. It also depends on the number of hours and mammograms performed by radiographers per week.

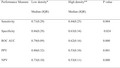

## Introduction

Breast cancer is the most common type of cancer in women worldwide.^1^ Early detection is key to decreased morbidity and mortality with dedicated screening programmes available in many countries worldwide including, Australia,^2^ the United States,^3^ and the United Kingdom.^4^ Routine mammography is the gold standard imaging method used to detect breast cancer and has been shown to contribute to at least a 30% reduction in the number of deaths from breast cancer in patients aged over 50 years.^5^ However, 2D mammography has limitations including false negatives which have been reported to account for 10% to 30% of missed breast cancers. Using 2D mammography 80% of woman recalled for additional views typically have normal outcomes.^6^

The radiologists’ ability to correctly interpret mammograms is strongly influenced by key personal characteristics including age, academic qualification, number of years since qualification,^7^ fellowship training,^8^ and workload.^9^ In the screening setting, the large number of women screened and the required speed of reading may also lead to less effective reporting due to fatigue and eye strain.^10^

Patient related factors may also affect the radiologists’ ability to interpret mammograms. Among the most important factor is breast density, mainly because women with higher breast density are more susceptible to developing breast cancer than women with less dense breasts.^11^ Higher breast density also results in less visibility (masking) of breast lesions in 2D mammography due to the low contrast between cancer and dense breast tissues.^12^

It has been reported that double reading screening mammograms increases cancer detection and decreases mortality from breast cancer.^13^ Double reading typically means that the same mammogram is interpreted by two radiologists,^14^ however, the high workload of radiologists has seen the evolution of the concept of a ‘skill mix’ in which radiographers contribute to image reporting as double readers.^15^ This concept has been used in the UK to reduce the radiologists’ workload by training radiographers to read mammograms in many screening units within the National Health Service Breast Screening Programme (NHSBSP).^16^

Several studies have assessed the diagnostic performance of radiographers in reading mammograms.^1^, ^15^, ^17^ In general, the use of radiographers as second readers has been shown to support the increase in the number of detected cancers afforded by double reading.^13^, ^18^, ^19^ However, no current studies have been found that assessed radiographers’ ability to interpret mammograms of differing breast density nor key radiographer demographics that may influence their ability to report on mammograms accurately. The aim of the current study is to measure Jordanian radiographers’ performance in interpreting mammograms and to compare performance measures in cases of differing breast density. This study also aims to examine key demographic factors that may influence their performance.

## Materials and Methods

Ethical approval was obtained through the Human Research Committee at Jordan University of Science and Technology (approval number: 470‐2020). Written informed consent was obtained from each radiographer before their participation.

### Cases

The study consisted of 30 screening cases acquired using computed radiography (CR), the most common mammography units available in Jordanian Hospitals. Each case comprised four routine digital mammograms (cranio‐caudal (CC) and medio‐lateral oblique views (MLO)) for both breasts. The images were selected by an experienced radiologist who had more than 20 years of experience in reading mammograms. In order to achieve the study aims, the radiologist was asked to select cases with different diagnostic outcomes. Of the selected images, 15 were normal as confirmed by a 2‐year follow up examination and 15 had a biopsy proven malignant lesions.

Cases were additionally purposively selected according to mammographic breast density and assigned a density category using the American College of Radiology (ACR) Breast Imaging‐Reporting and Data System (BI‐RADS) 5th edition.^20^ This classification system consists of four categories, ‘a. the breasts are almost entirely fatty, b. there are scattered areas of fibroglandular density c. the breasts are heterogeneously dense, which may obscure small masses and d. the breasts are extremely dense, which lowers the sensitivity of mammography’. BI‐RADS density scoring was confirmed by two other radiologists and in case of disagreement; the majority rating (two of three readers) was used. Cases that scored BI‐RADS a and b were considered as low mammographic breast density (*n* = 14), while cases of BI‐RADS c and d were considered high‐mammographic breast density (*n* = 16) ^20^. Low mammographic density cases included seven normal and seven abnormal mammograms, while high‐mammographic density cases included eight normal and eight abnormal mammograms.

### Participants and study design

This study was conducted in North Jordan. All radiographers working as mammographers at the four main public and private hospitals were invited to participate. Twelve female radiographers aged between the 20 and 50 agreed to participate; none had formal training in reading mammography images. The radiographers were asked to read images displayed on an 8‐megapixel (MP) workstation calibrated according to the Digital Imaging and Communications in Medicine (DICOM) standard. Radiographers were trained to use the available image processing tools including magnification, windowing and panning, and were given unlimited time to read and score all images. Each radiographer was asked to determine if each image was normal or needed to be recalled and to assign a BI‐RADS assessment category 1–5,^21^ where a score of 1 represents ‘no significant abnormality’, 2 is ‘benign finding’, 3 is ‘indeterminate/equivocal finding’, 4 is ‘suspicious findings of malignancy’ and 5 is ‘malignant findings’.

### Data analysis

Statistical analysis was performed with Statistical Package for Social Sciences (SPSS) 26.0 software. Frequency and percentage analysis were carried out to investigate the descriptive characteristics of study sample. The performance of each radiographer was assessed using; sensitivity, specificity, positive predictive value (PPV), negative predictive value (NPV) and area under the receiver operating characteristic curve (ROC, AUC).

Non‐parametric hypothesis tests were performed throughout the whole data analysis after performing Kolmogorov–Smirnov and Shapiro–Wilk tests to check for normality. Mann Whitney *U* test was applied for the comparison between groups, median and interquartile range values were reported. A *P* value of ≤0.05 was considered statistically significant. The sample size used in this study was able to detect a difference of 0.06 of each performance measure at 80% power. Gender and training background were excluded from the analysis because all readers were female, and none had formal training in image interpretation.

## Results

Table 1 reports the socio‐demographic and professional characteristics of study participants. All 12 participating radiographers were females, 7 (58.3%) were between the age of 20 to 30 and the same percentage 58.3% work in public hospitals and teaching hospitals. More than half (58.3%) of the participants had 1‐ to 5‐year experience in breast imaging. In relation to workload, half of the radiographers worked in mammography imaging ≤20 hours per week and 41.7% performed ≥20 mammography cases per week. All participating radiographers had no previous training in reading mammography images.

**Table 1:** Socio‐demographic and professional characteristics of study participants (*n* = 12).

Characteristic	No (%)
Age	
20–30	7 (58.3)
≥30	5 (41.7)
Section	
Public	7 (58.3)
Private	5 (41.7)
Hospital	
Teaching	7 (58.3)
Non‐teaching	5 (41.7)
Level of Education/Radiography	
Bachelor	8 (66.7)
Diploma	4 (33.3)
Years working in mammography	
<1	3 (25.0)
1–5	7 (58.3)
6–10	2 (16.7)
Hours working on mammography per week	
≤20	6 (50.0)
>20	6 (50.0)
Cases performed per week	
<20	7 (58.3)
≥20	5 (41.7)
Computed (CR) or Digital radiography (DR) acquisition	
CR	8 (66.7)
DR	4 (33.3)

Table 2 reports the performance measures of each radiographer. The ranges of sensitivity, specificity, ROC, PPV and NPV were 0.33–0.80, 0.33–0.93, 0.57–0.80, 0.50–0.88 and 0.50–0.71 respectively.

**Table 2:** Performance measures of study participants for all cases.

Reader	Sensitivity	Specificity	ROC AUC	PPV	NPV
1	0.46	0.93	0.71	0.88	0.64
2	0.53	0.46	0.59	0.50	0.50
3	0.40	0.73	0.58	0.60	0.55
4	0.53	0.80	0.78	0.73	0.63
5	0.46	0.80	0.73	0.70	0.60
6	0.53	0.86	0.74	0.80	0.65
7	0.66	0.73	0.70	0.71	0.69
8	0.33	0.80	0.58	0.63	0.55
9	0.80	0.46	0.68	0.60	0.70
10	0.80	0.33	0.62	0.55	0.63
11	0.73	0.60	0.81	0.65	0.69
12	0.73	0.67	0.75	0.69	0.71
Mean	0.58	0.68	0.69	0.67	0.63

ROC AUC, area under receiver operating characteristic curve; PPV, positive predictive value; NPV, negative predictive value.

Table 3 shows the difference in radiographers' performance in low compared to high breast density cases. All performance measures were significantly higher in low compared to high breast density mammograms (*P* value ranges from 0.000–0.024).

**Table 3:** Difference in performance measures between cases with different density.

Performance Measure	Low density* Median (IQR)	High density** Median (IQR)	*P* value
Sensitivity	0.71 (0.29)	0.44 (0.25)	0.004
Specificity	0.86 (0.29)	0.63 (0.34)	0.024
ROC AUC	0.79 (0.09)	0.62 (0.16)	0.000
PPV	0.80 (0.32)	0.53 (0.10)	0.001
NPV	0.73 (0.10)	0.53 (0.11)	0.000

IQR, Interquartile range; ROC AUC, area under receiver operating characteristic curve; PPV, positive predictive value; NPV, negative predictive value.

* BI‐RADS a and b.

** BI‐RADS c and d.

As indicated in Table 4, radiographers who had greater years of experience in mammography, who worked for longer hours and who perform more cases per week had significantly higher mean PPV compared with radiographers with less years of experience, less work hours and less number of cases (*P* value = 0.023, 0.01, 0.017 respectively). The results also demonstrated that the radiographers who work >20 hours in mammography weekly and who perform ≥20 mammograms per week had significantly higher ROC AUC (*P* value = 0.001 and 0.004 respectively).

**Table 4:** Difference in performance measures according to demographic and professional characteristics. P value, median (IQR) are reported.

Readers/groups	Sensitivity	Specificity	ROC AUC	PPV	NPV
Age					
<30	0.66 (0.33)	0.68 (0.72)	0.68 (0.17)	0.63 (0.09)	0.69 (0.15)
≥30	0.53 (0.21)	0.80 (0.33)	0.73 (0.09)	0.73 (0.22)	0.63 (0.03)
*P* value	0.68	0.336	0.348	0.084	0.946
Section					
Public	0.53 (0.34)	0.80 (0.40)	0.71 (0.12)	0.70 (0.25)	0.63 (0.05)
Private	0.66 (0.37)	0.73 (0.13)	0.70 (0.20)	0.65 (0.08)	0.69 (0.15)
*P* value	0.864	0.707	0.860	0.717	0.694
Hospital					
Teaching	0.53 (0.34)	0.80 (0.40)	0.71 (0.12)	0.70 (0.25)	0.63 (0.05)
Non‐teaching	0.66 (0.37)	0.73 (0.13)	0.70 (0.20)	0.65 (0.08)	0.69 (0.15)
*P* value	0.864	0.707	0.694	0.717	0.694
Education					
Bachelor	0.53 (0.30)	0.73 (0.23)	0.71 (0.17)	0.67 (0.17)	0.65 (0.14)
Diploma	0.67 (0.32)	0.63 (0.44)	0.71 (0.13)	0.65 (0.16)	0.63 (0.08)
*P* value	0.322	0.285	0.702	0.587	0.691
Years as a mammographer					
<1	0.66 (0.47)	0.73 (0.47)	0.62 (0.12)	0.63 (0.16)	0.63 (0.14)
1–5	0.53 (0.27)	0.67 (0.34)	0.73 (0.19)	0.65 (0.13)	0.63 (0.1)
6–10	0.50 (.)*	0.90 (.)*	0.73 (.)*	0.84 (.)*	0.65 (.)*
*P* value	0.750	0.198	0.389	**0.023**	0.940
Hours/week					
≤20	0.60 (0.42)	0.60 (0.32)	0.61 (0.11)	0.60 (0.11)	0.59 (0.15)
>20	0.53 (0.27)	0.80 (0.22)	0.75 (0.06)	0.72 (0.14)	0.65 (0.07)
*P* value	0.893	0.066	**0.001**	**0.010**	0.211
Cases/week					
<20	0.53 (0.40)	0.73 (0.34)	0.62 (0.12)	0.60 (0.15)	0.60 (0.14)
≥20	0.53 (0.24)	0.80 (0.26)	0.75 (0.07)	0.73 (0.17)	0.65 (0.06)
*P* value	0.784	0.153	**0.004**	**0.017**	0.124
Equipment					
CR	0.47 (0.39)	0.77 (0.32)	0.63 (0.15)	0.62 (0.16)	0.60 (0.14)
DR	0.60 (0.25)	0.70 (0.31)	0.72 (0.13)	0.70 (0.15)	0.64 (0.08)
*P* value	0.341	0.692	0.187	0.787	0.588

IQR, Interquartile range, ROC AUC, area under receiver operating characteristic curve, PPV, positive predictive value, NPV, negative predictive value, CR, computed radiography, DR, digital radiography.

Numbers in bold represent a significant difference.

* This group has only 2 participants.

## Discussion

With the introduction of breast screening programmes and the associated increase in the number of women undergoing mammography, a high demand has been placed on radiologists to perform screen reads. In particular the need in many screening services to double read cases and have a third reader if there is discordance has created workload issues.^22^ An important measure that needs to be considered to address this workload issue is role extension for radiographers which has been used in the UK^16^ and suggested in other counties such as Australia.^23^ Previous work has demonstrated that radiographers sensitivity and specificity in reading mammograms was comparable to that of radiologists^17^, ^24^, ^25^ and that the addition of radiographers as a second reader can also contribute positively by detecting more cancers in the screening setting.^13^, ^18^, ^19^ It has been reported that the contribution of a radiographer as a double reader resulted in the detection of 9% more cancers compared with a single reading by a radiologist.^18^

In Jordan, heightened public awareness associated with the development of the Jordanian breast screening programme has increased compliance with screening guidelines. This has resulted in a higher demand for breast screening services including mammography readers. Recently, it has been reported that the shortage of specialised radiologists is one of the main workforce gaps in mammography screening in Jordan ^26^. This suggests that the same concept of a ‘skill mix’ can potentially be used as a solution to the subsequent increase in radiologists’ workload associated with the higher number of women screened women. However, other contributing factors such as the education and training of Jordanian radiographers must be considered before the establishment of a double reading strategy. This requires the assessment of radiographer’s current performance in reading mammograms as a first step towards future recommendations.

The overall results of the current study showed a relatively low to medium mean sensitivity, specificity, ROC AUC, PPV and NPV of 0.58, 0.68, 0.69, 0.67 and 0.63, respectively. However, the results were heterogeneous among radiographers and a wide variation, particularly in sensitivity and specificity values ranging from 0.33–0.80 and 0.33–0.93 respectively were seen. These results are comparable with previous studies which also showed a wide range of sensitivity (61–89%) and specificity (45–97%) among radiographers.^24^ While the performance measures of participating radiographers in this study are lower than those reported in other studies ^1^, ^17^, ^24^, ^27^ it must be acknowledged that some of these studies^1^, ^23^ included radiographers who had higher (up to 44 years) experience in mammography compared to 10 or less years of experience in this study. It must also be noted that all mammograms used in the current study were acquired using a CR unit due to the higher availability of CR systems in Jordanian hospitals. It has previously been reported that CR systems have a lower cancer detection rate than DR systems.^28^ The low level of performance in the current research might also be attributed to differences in radiographers’ training. All participating radiographers had no previous training in mammographic image interpretation. Previous work reported that dedicated and self‐study training programmes may improve the performance of radiographers not only in detecting cancer, but also in identifying benign lesions and reducing the number of false positive.^6^, ^23^ An increase in Jordanian radiographer performance may be evidenced in future studies with formalised screen reading training and assessment.

While radiographers in some countries receive postgraduate formal training in image interpretation such as in the United Kingdom (UK) ^29^ there is no similar approach in Jordan. Radiographers typically gain image interpretation skills through individual efforts and by communication with radiologists and other radiographers during practice.

After dividing the cases into high‐ and low‐breast density categories, our results showed that even without formal training radiographers may have comparable reporting skills to radiologists in low mammographic density breasts reporting a mean sensitivity, specificity, ROC AUC, PPV and NPV of 0.70, 0.80, 0.79, 0.81, 0.73 respectively. This has an important implication on the planning of radiographer's contribution to image interpretation where they may potentially be recruited to read cases with low mammographic density which may relieve radiologists’ workload and free up radiologists for more difficult tasks (which could include reporting mammograms with high‐mammographic density). This has been introduced elsewhere as a more cost‐effective scenario than reading all mammograms by either the radiographer or radiologist.^30^ Providing formal training programmes in image interpretation focusing on high‐mammographic density cases could alternatively be considered for radiographers who wished to become dedicated screen readers.

The results regarding the association between radiographer demographics and performance showed higher PPV for radiographers who had 6–10 years of experience compared with less experienced radiographers. Also, higher PPV and ROC AUC for radiographers who worked for more than 20 hours and who performed 20 or more cases per week compared to those who had less workload in terms of number of hours or cases per week. In line with the results of the current work, it has previously been reported that the most experienced radiology readers have the highest PPV^31^ which can be explained by the cumulative exposure to normal radiographic features of mammograms and being more able to distinguish abnormal findings. Similarly previous work also found that the performance of radiologists can also be affected by their years of experience and number of reading hours per week.^32^

This study has some limitations. First, the sample size and the number of readers were relatively small and unlike other published studies participating radiographers were not trained and assessed in screen reading as this was not within the aims of the study. Also, location sensitivity was not calculated in this study as the radiographers were not asked to localise the detected lesions due to time considerations. All images included in the current study were acquired using CR, however, not all participating radiographers were familiar with CR acquired images which may have contributed to the variation in radiographer performance.

In conclusion, the findings of this pilot study suggest that radiographers working in breast imaging have an inherent skill set that could be capitalised on to support the radiology workforce in Jordan. A more extensive study is required to determine if Jordanian radiographers are capable of successfully taking part in breast screen reading. The lower performance measures in radiographer interpreted high‐mammographic breast density cases emphasises the importance of any training programme providing education that focused on image interpretation skills in the dense breast.

## Conflict of Interests

The authors declare that there is no conflict of interest.
